# Freeze-Dissolving Method: A Fast Green Technology for Producing Nanoparticles and Ultrafine Powder

**DOI:** 10.1021/acssuschemeng.2c02270

**Published:** 2022-06-09

**Authors:** Qiushuo Yu, Yingchen Wang, Jiaqi Luo, Huaiyu Yang

**Affiliations:** † School of Chemical Engineering, Northwest University, Xi’an, Shaanxi 710069, China; ‡ Department of Chemical Engineering, Loughborough University, Loughborough LE11 3TU, United Kingdom

**Keywords:** Freeze-dissolving, Freeze-drying, Nanoparticles, Ultrafine powder, Sustainable, Ice template

## Abstract

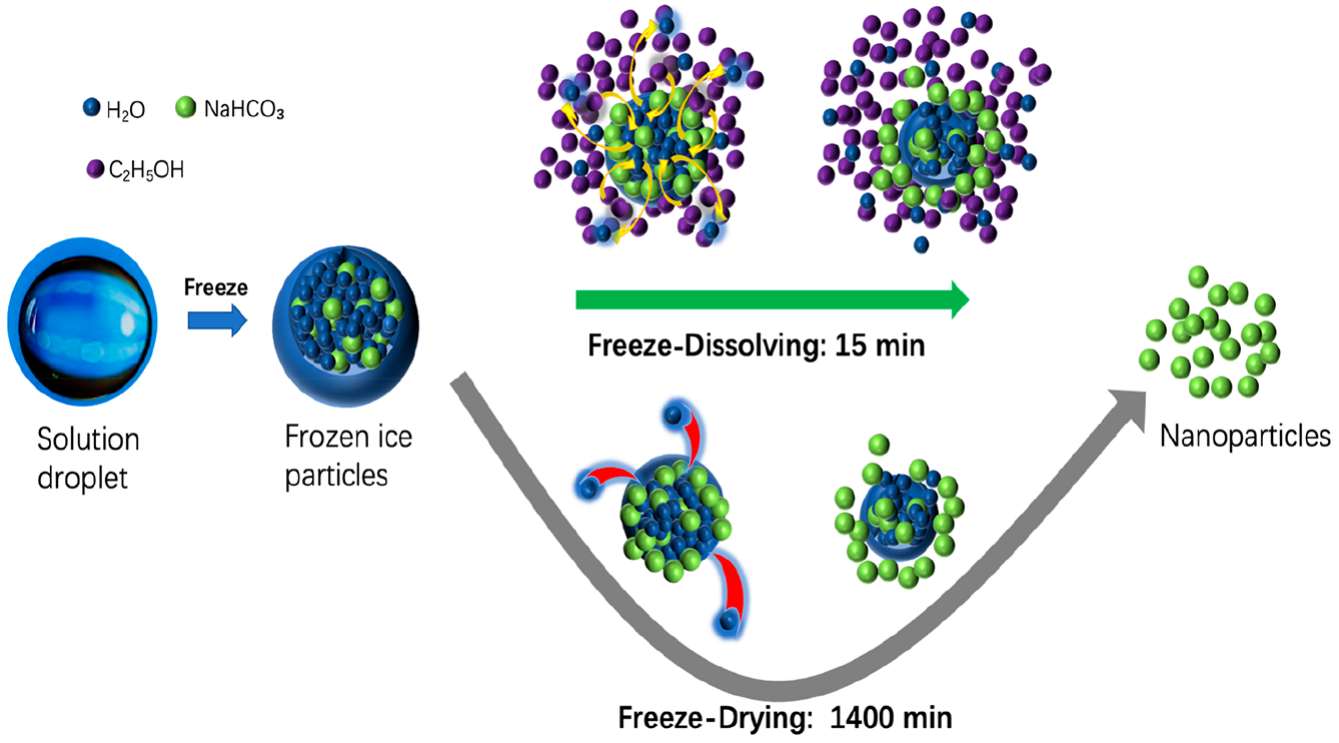

A new technology, a freeze-dissolving method, has been developed to isolate nanoparticles or ultrafine powder and is a more efficient and sustainable method than the traditional freeze-drying method. In this work, frozen spherical ice particles were produced with an aqueous solution of sodium bicarbonate or ammonium dihydrogen phosphate at various concentrations to generate nanoparticles of NaHCO_3_ or (NH_4_)(H_2_PO_4_). The freeze-drying method sublimates ice, and nanoparticles of NaHCO_3_ or (NH_4_)(H_2_PO_4_) in the ice templates remain. The freeze-dissolving method dissolves ice particles in a low freezing point solvent at temperatures below 0 °C, and then, nanoparticles of NaHCO_3_ or (NH_4_)(H_2_PO_4_) can be isolated after filtration. The freeze-dissolving method is 100 times faster with about 100 times less energy consumption than the freeze-drying method as demonstrated in this work with a much smaller facility footprint and produces the same quantity of nanoparticles with a more uniform size distribution.

## Abstract 2

A novel freeze-dissolving method for nanoparticles or ultrafine powder production is much more efficient and sustainable than the traditional freeze-drying method.

## Introduction

Nanoscale material and ultrafine powders have increasing applications, such as in environmental and sustainable areas,^1−3^ drawing significant interests due to their large specific surface areas^4−6^ and high reaction activities.^7−9^ Freeze-drying technology has been often used to produce nanoparticles and ultrafine particles.^10−13^ For the freeze-drying technology, the first step is a freezing process to freeze water with target molecules, particles, or materials inside. During the fast-freezing process, water in the aqueous solution is solidified in a short period, forming a network of ice crystals, also known as freeze-casting or ice templating. The network of ice crystals drives the target solute molecule or building blocks to form a nanoscaffolding structure, forming nanoporous or microporous products.^14−16^ The freezing step determines the morphology of the ice crystal template and scaffolding structure by the freezing conditions^17−19^ and, correspondingly, the crystalline structure of the target material products within the ice scaffolding or templates.^20,21^

The second step is a drying process to remove water as ice scaffolding by sublimation. During the drying process, the frozen ice leaves, and the target molecules, materials, or particles remain inside the ice. The freeze-casted nanoparticles or porous materials can be recovered with the same structure and properties inside the ice. The low temperature used in the drying process results in slow sublimation rates, and typical pharmaceutical products have batch drying times in the order of days. The manufacturing rates of such batch-based processes are limited by low freeze-drying rates and long cycle operational periods. A larger freeze-dryer can mitigate some of these disadvantages. However, a longer time is required to achieve ideal vacuum conditions, and the pressure and temperature are less uniform across the chamber, which can impact product quality.^22,23^ The drying process requires huge energy consumption due to the low temperature and the vacuum system.^24^

In this work, a freezing-dissolving technology was for the first time developed as a fast, simple, and sustainable method as an alternative to the freeze-drying technology. The first step of freeze-dissolving is same as freeze-drying, which is freeze-casting to produce ice with the target materials inside and form a target structure with ice-scaffolding. The second step of freeze-dissolving is to dissolve the ice in another solvent with a low freezing point at a low temperature, such as below 0 °C. This second solvent is an antisolvent to the target materials but miscible with water, such as ethanol. Therefore, the ice scaffolding will be quickly dissolved in the second solvent, with only the target materials left as a solid phase in the solution, and the structure of target materials formed inside the ice will be preserved. Sodium bicarbonate,^25−27^ baking soda, and ammonium dihydrogen phosphate,^28^ fire extinguishing agents,^29,30^ are soluble in water and insoluble in ethanol. NaHCO_3_ or (NH_4_)(H_2_PO_4_) in water with different concentrations was used to produce nanoparticles by the freeze-dissolving method, which were compared with nanoparticles generated by freeze-drying, respectively. This new freeze-dissolving technology with a simple facility and much less energy consumption greatly shortens the isolation period compared with the freeze-drying technology, and moreover, with freeze-dissolving technology, nanoparticle products with better quality can be produced.

## Experimental Section

### Materials

Sodium bicarbonate, NaHCO_3_, and ammonium dihydrogen phosphate, (NH_4_)(H_2_PO_4_), were purchased from Tianjin Baishi Chemical Co., Ltd. (purity > 99.5%). Ethanol was purchased from Tianjin Damao Chemical Reagent Factory (purity > 99.7%), and acetone (purity > 99.5%) was purchased from Tianjin KeMiOu Chemical Reagent Factory. All chemicals were used without further purification. Distilled deionized water (conductivity < 0.5 μS/cm) was used.

### Experiment

**Figure 1:**
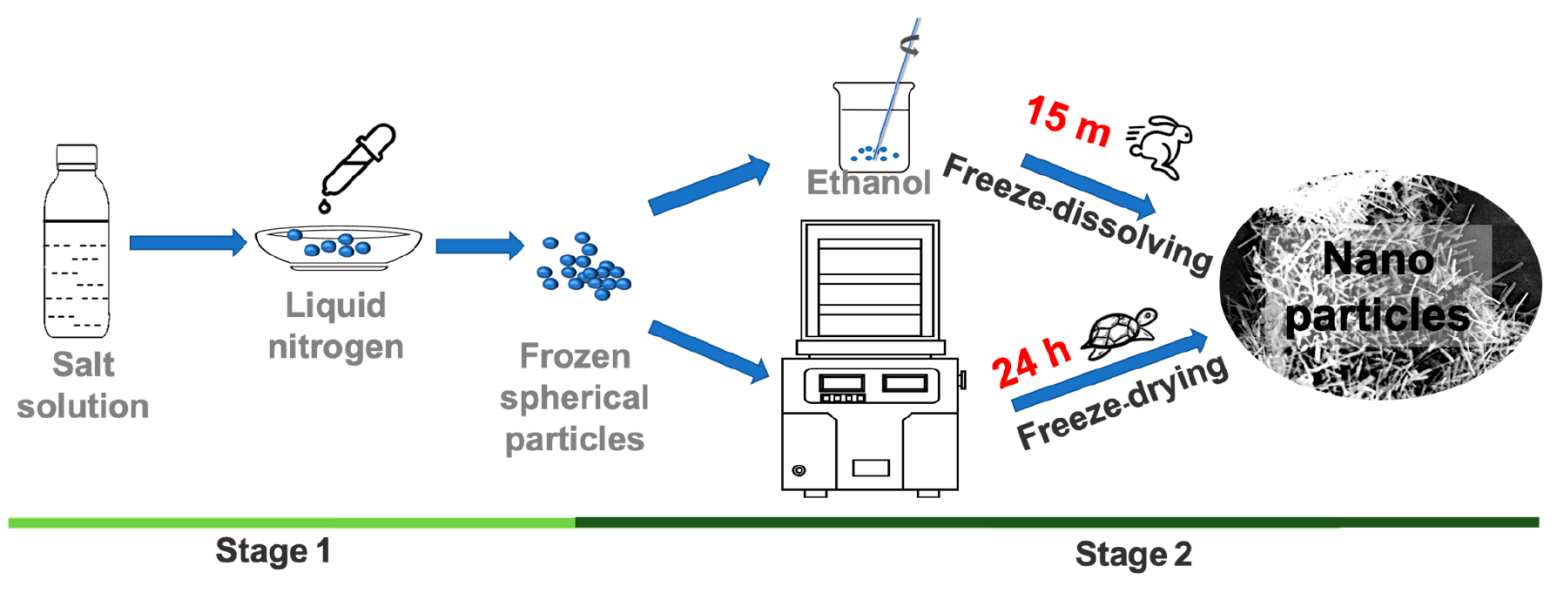
Schematic diagram of experimental setup for the freeze-dissolving method (top) and the freeze-drying method (bottom).

The nanoparticles of NaHCO_3_ were prepared by two steps, shown in Figure 1. In the first step, sodium bicarbonate was dissolved in water at 35 °C, with different concentrations of 0.2, 0.6, 1.0, and 1.5 g of NaHCO_3_ in 1.0 g of water. The solution was transferred by a pipette into an iron plate containing about 10 mL of liquid nitrogen to form frozen particles (Stage 1). Different sizes of the frozen spherical particles were prepared by dropping large, medium, and small droplets into the liquid nitrogen to form particles with average masses of 0.0135, 0.0439, and 0.0706 g, respectively. All droplets quickly sank into the liquid nitrogen. The average mass was calculated based on 200 frozen spherical particles of each size, with less than 10% uncertainty, and the corresponding average volumes were estimated to be 0.01, 0.04, and 0.06 cm^3^, respectively.

In Stage 2, two methods were used to collect the nanoparticles separately: (i) The frozen particles with similar sizes were dried for 24 h from −30 to −50 °C in a freeze-dryer (FD-1A-50, Beijing Boyikang Experimental Instrument Co., Ltd., China). (ii) The frozen particles were poured into ethanol (with a mass seven times the mass of the water for preparing frozen particles) at −10 °C in a beaker, which was in a −10 °C water bath for 5 min with continuous stirring. The circulating liquid used in the water bath was a mixture of water and ethanol with a freezing point much lower than −10 °C. The ice in the frozen particles was dissolved, and the nanoparticles in the solution were filtered with membrane PTFE (Whatman) of a 1 μm pore size with a dead-end vacuum filtration method for 5 min. The particles were quickly packed, forming “filter cake” on the membrane, and the liquid flow rate was above 6 mL/min. After filtration, the particles on the membrane were dried for 5 min with additional 20 min to ensure being totally dried.

A cooling crystallization experiment with NaHCO_3_ was performed by cooling a 0.10 g/g sodium bicarbonate aqueous solution from 45 to 0 °C with stirring until crystallization, and then, the particles were filtered.

Solubility of NaHCO_3_ in a water + ethanol binary solvent at 273.15–313.15 K was determined by a gravimetric method (Supporting Information), which provides guidance for designing the experimental conditions for dissolving the frozen particles.

### Characterization

The morphologies and particle sizes of the samples were characterized by a laser particle size analyzer (Zetasizer Nano ZSP, Malvern) and scanning electron microscope (TM3000, Hitachi, Ltd., Japan). Powder X-ray diffraction of the product samples of 30 mg was determined by a Smartlab powder diffractometer with radiation (1.5406 Å). About 0.05 g of nanoparticle products was added to an acetone solution, which was then dispersed in an ultrasonic disperser for 5–10 min. Three samples based on the same experimental conditions were prepared. Each sample was measured three times by a nanolaser particle sizer (Malvern), and the test range was focused from 10 nm to 10 μm.

## Results and Discussions

### Case of Sodium Bicarbonate

**Figure 2:**
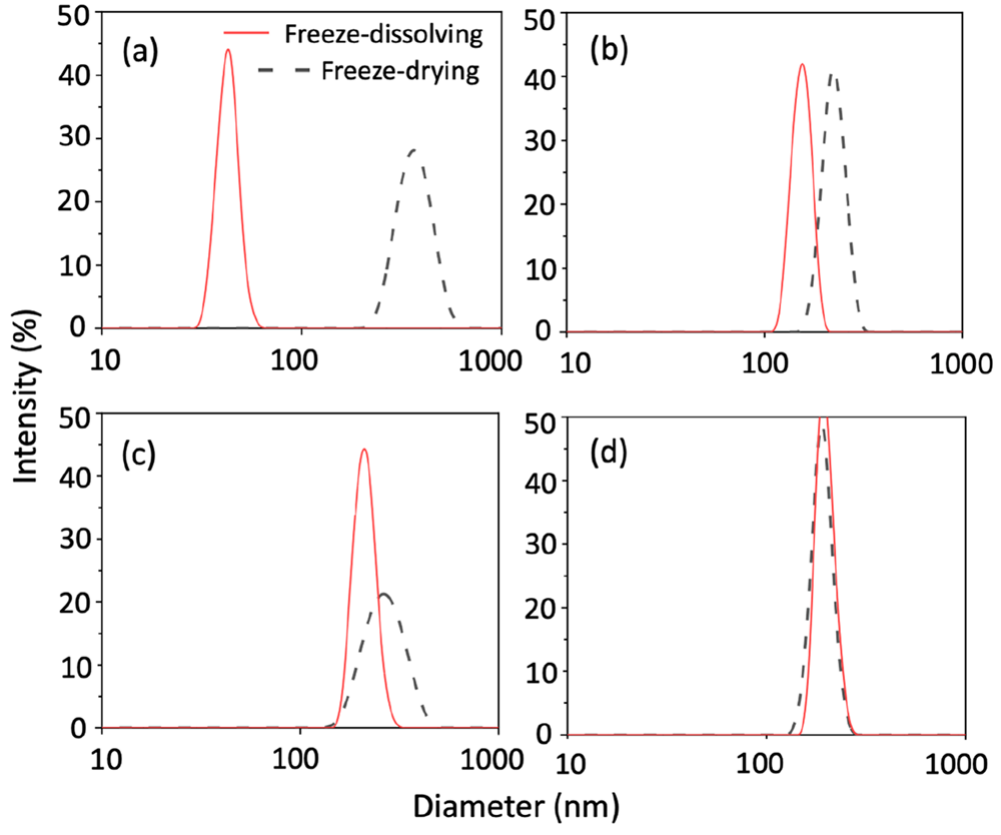
Particle size distribution of NaHCO_3_ nanoparticles with different concentrations: (a) 0.02, (b) 0.06, (c) 0.10, and (d) 0.15 g/g with the freeze-dissolving and freeze-drying methods.

**Figure 3:**
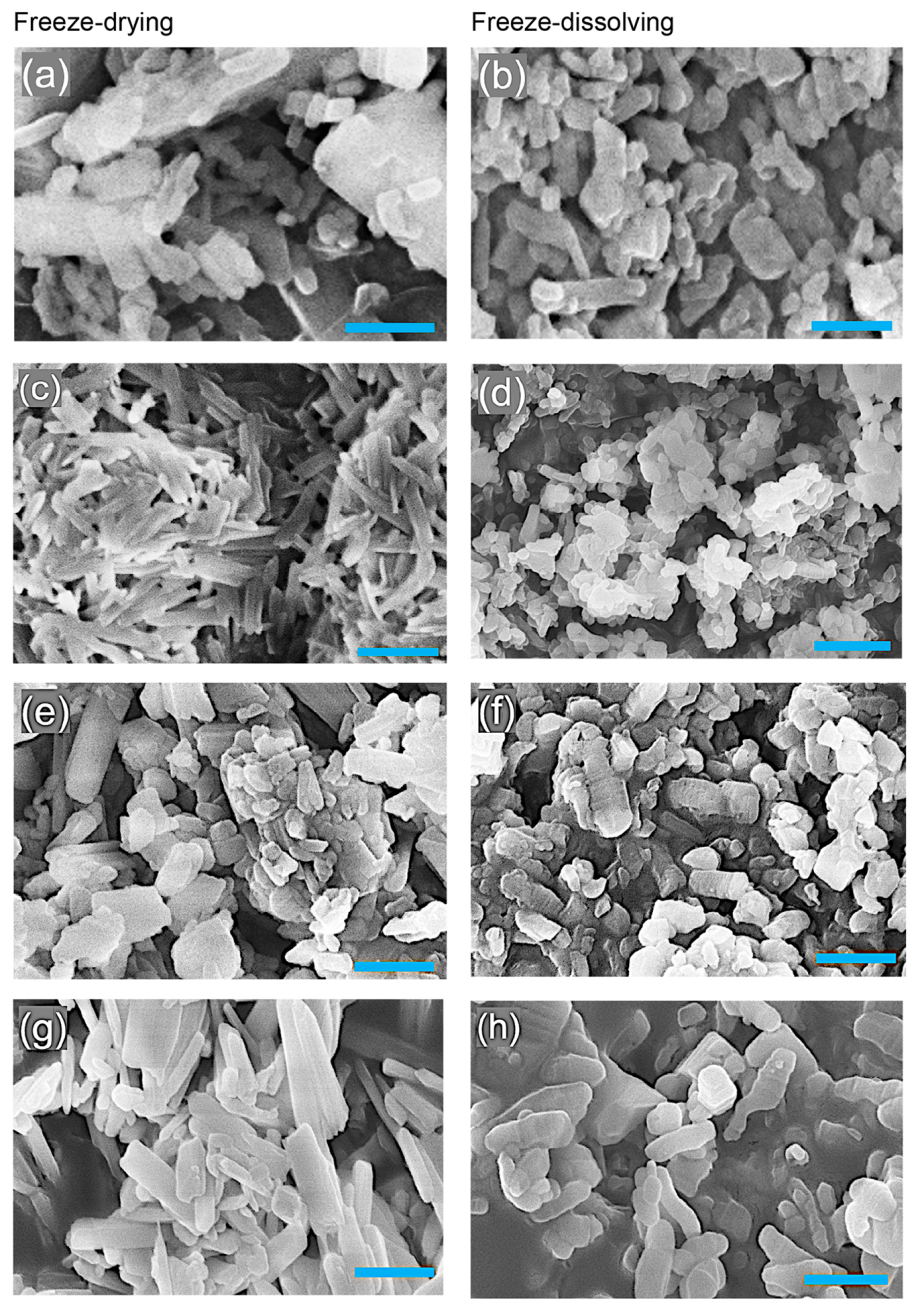
SEMs of crystalline nanoparticles (average size of 0.04 cm^–3^) of NaHCO_3_ by the freeze-drying (a, c, e, g) and freeze-dissolving (b, d, f, h) methods with concentrations of 0.02, 0.06, 0.10, and 0.15 g/g from top to bottom, respectively. Scale bar: 500 nm.

The nanoparticles of NaHCO_3_, obtained by the freeze-drying and freeze-dissolving methods, were all below 1 μm, with the average diameter in the range of 50–500 nm, shown in Figure 2. The particles obtained by the freeze-dissolving method were overall smaller than those obtained by the freeze-drying method, where the frozen spherical particles with equal diameters were used in both methods. At a very low concentration, 0.02 g NaHCO_3_/g water, the nanoparticles obtained by the freeze-dissolving method were more than five times smaller than those obtained by the freeze-drying method. With increases in the concentrations, the differences became smaller. With a concentration of 0.15 g NaHCO_3_/g water, the sizes of nanoparticles obtained by the two methods were similar. The SEM images in Figure 3 are in agreement with the particle size distributions. The nanoparticles by the freeze-drying method were formed with a 3D scaffolding structure with some agglomerations which were also reported in other systems with water/ice templates,^21^ while the textures of the nanoparticles obtained by the freeze-dissolving method appeared more flocculent.

**Figure 4:**
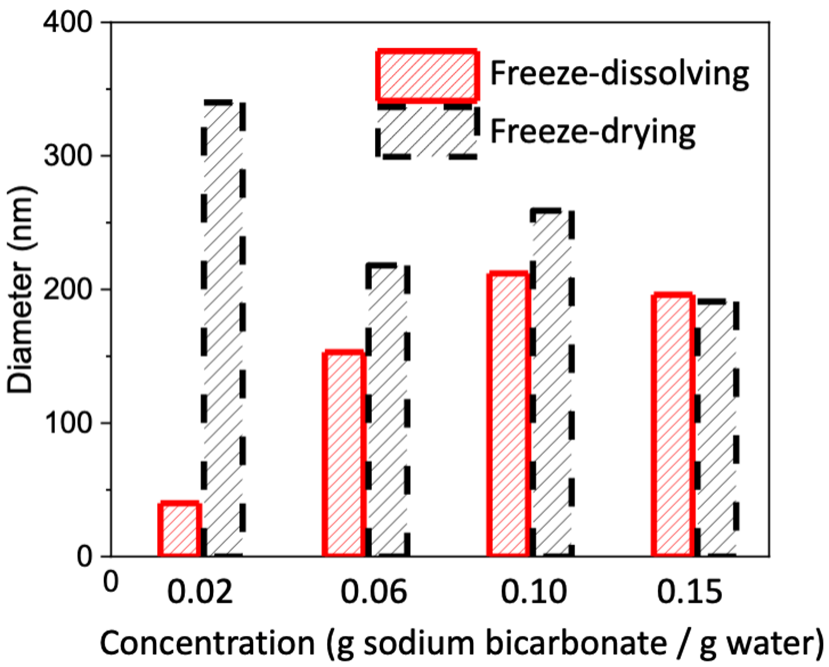
Average sizes of nanoparticles obtained by the freeze-drying and freeze-dissolving methods with different concentrations of NaHCO_3_.

The average size of the nanoparticles with the freeze-dissolving method tended to increase with an increase in the concentration of the sodium bicarbonate for preparing the frozen spherical particles, as shown in Figure 4, while there were variations of the average particle sizes with the freeze-drying method with different concentrations of NaHCO_3_.

There is no requirement of vacuum for the freeze-dissolving method, and therefore, comparing with the freeze-drying method, the freeze-dissolving method provides a quick approach to make nanoparticles with a simple facility and much less energy consumption. Moreover, the freeze-dissolving method only took 5 min for dissolving, with an additional 5 min for filtration and 5 min drying in this work, which greatly reduced the process period, compared with an operation period of more than 24 h with the freeze-drying method. It is noted that the fast filtration and drying may be due to the small quantity of particles produced, and the efficiency will need to be further tested in larger scales.

Despite the influences of the freeze-drying and freeze-dissolving methods during the isolating process for the nanoparticles, the nanoparticles of NaHCO_3_ were formed in the process of preparing the frozen spherical particles (ice particles) in the first stage, dependent on the frozen rate that was determined by the droplets sizes. More experiments were designed to investigate the influence of the size of the frozen particles on the final size of the nanoparticles of NaHCO_3_.

**Figure 5:**
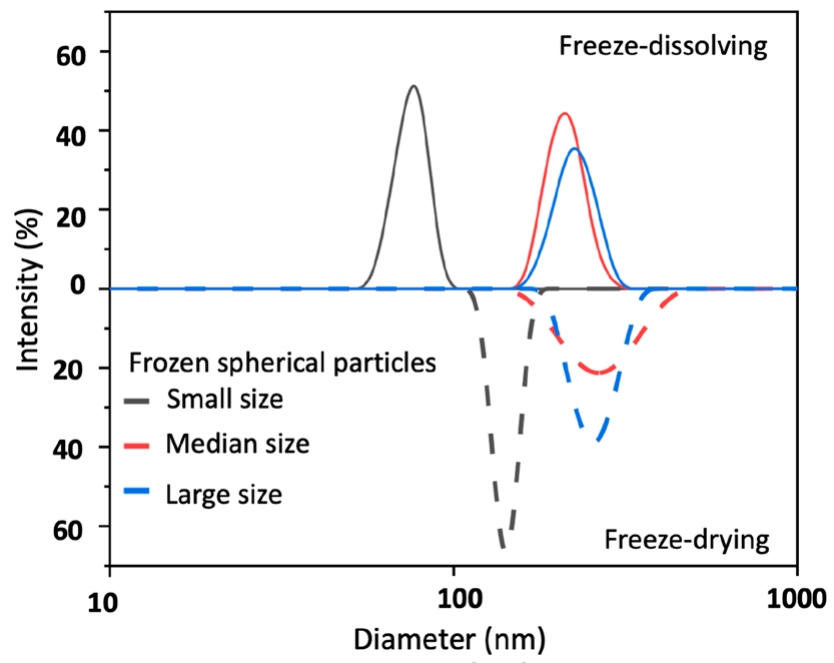
Particle size distributions of NaHCO_3_ nanoparticles isolated from frozen spherical particles with different average sizes of 0.01, 0.04, and 0.06 cm^3^ by the freeze-drying and freeze-dissolving methods. The concentration was 0.10 g/g for preparing the frozen spherical particles.

**Figure 6:**
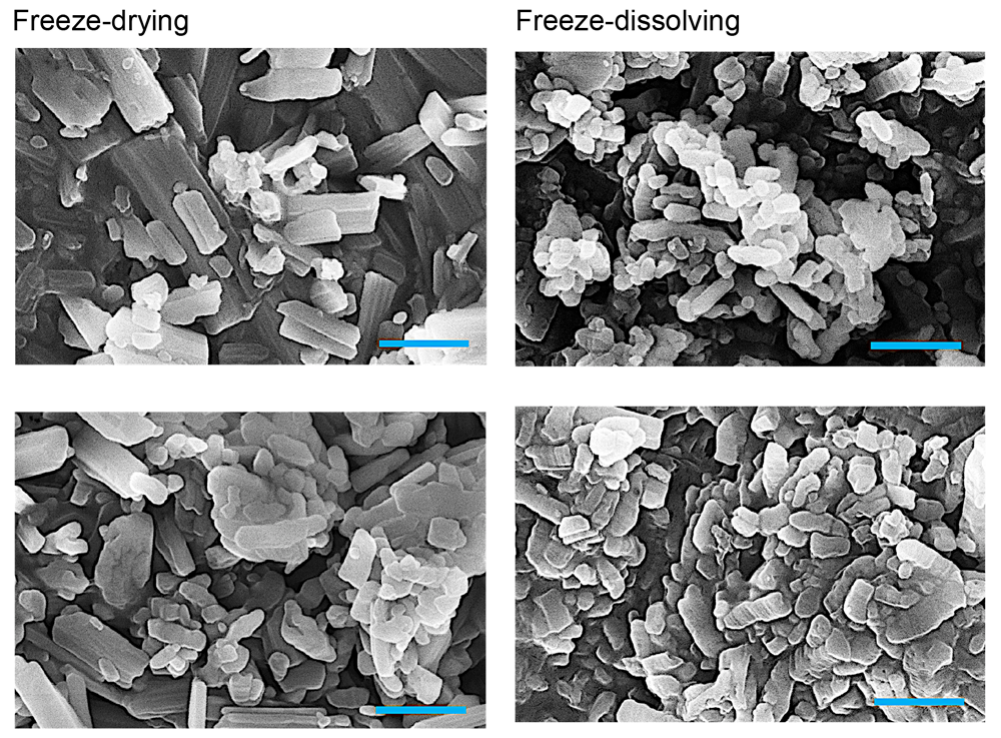
SEM images of NaHCO_3_ crystalline nanoparticles by the freeze-drying method (left) and freeze-dissolving method (right) with average sizes of 0.01 cm^3^ (top), 0.04 cm^3^ (same as (e) and (f) in Figure 3), and 0.06 cm^3^ (bottom) of the frozen spherical particles. The concentration was 0.10 g/g for preparing the frozen spherical particles. Scale bar: 500 nm.

Figure 5 shows that, with equal sizes of the frozen particles, the NaHCO_3_ nanoparticles obtained by the freeze-dissolving method were overall smaller than those obtained by the freeze-drying method, respectively. With smaller sizes of the frozen spherical particles, the nanoparticles obtained by the freeze-dissolving method were more than five times smaller than those obtained by the freeze-drying method. With a decrease in the sizes of the frozen particles, the differences became smaller, and the size distribution of the nanoparticles by the freeze-dissolving method were narrower than those by the freeze-drying method. Figure 6 shows a consistent trend. The small frozen particles with a shorter time to be frozen resulted in narrower size distributions due to less aggregations during the freezing process when the dissolved NaHCO_3_ solidified/nucleated. In addition, there were more NaHCO_3_ molecules in the middle and large size ranges of the droplets and frozen particles, leading to high chances to form larger aggregations (wider size distribution) during the freezing process. There was no obvious difference on the PXRD of these final products obtained by the same method, indicating a similar crystalline structure of these final products.

**Figure 7:**
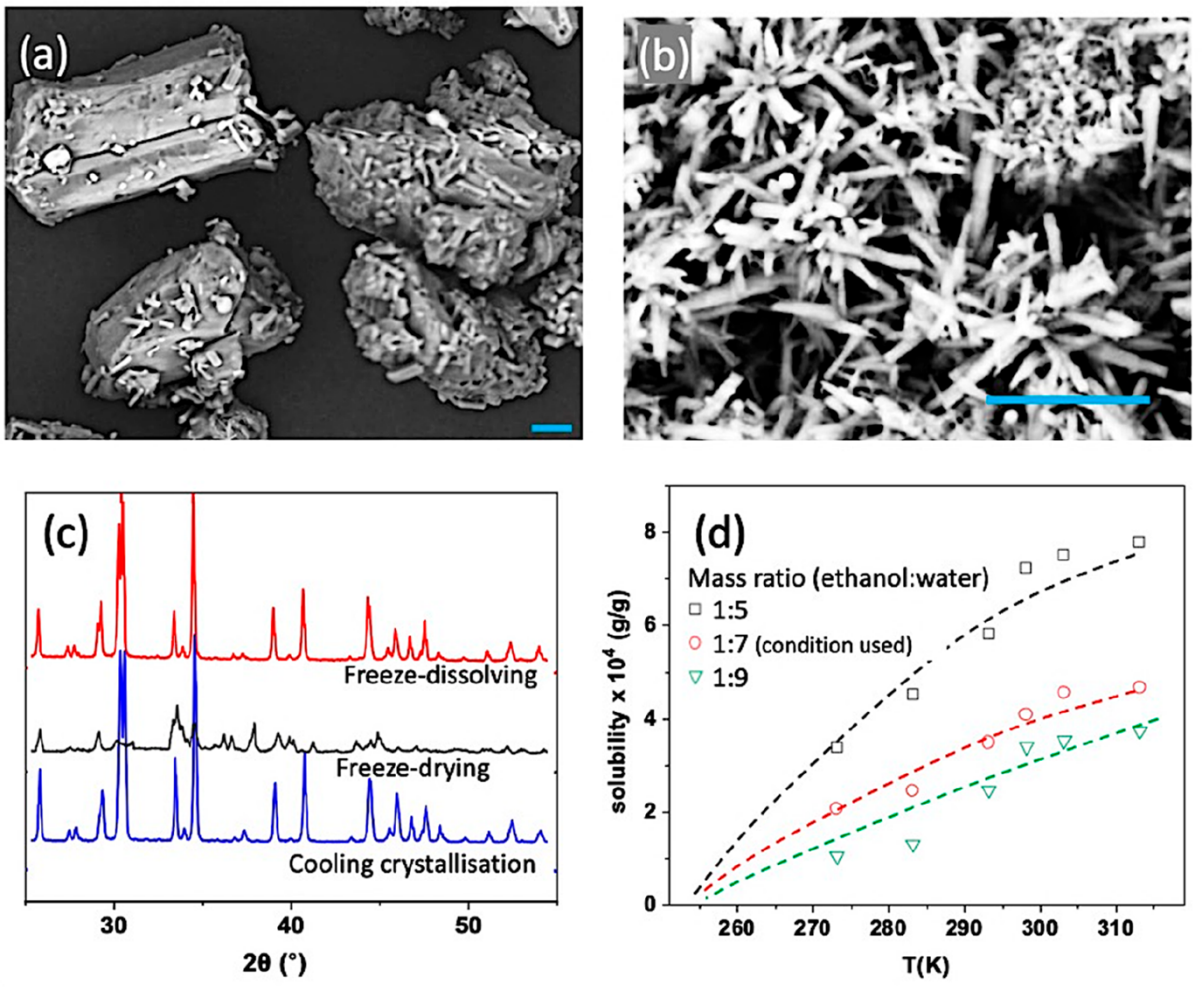
SEM of NaHCO_3_ particles of raw material (a) and obtained from fast cooling crystallization (b). Scale bar: 10 μm. Powder XRD spectra of sodium bicarbonate particles (c), obtained from the freeze-dissolving method (top), the freeze-drying method (middle), and cooling crystallization (bottom). Solubilities of sodium bicarbonate (d) in water and ethanol binary solvent at different temperatures.

With the same concentration of 0.10 g/g of NaHCO_3_ in the solution, the particles with average sizes of 3000 nm were obtained by fast cooling crystallization, shown in Figure 7(b), which were much smaller than the particles of the raw material, shown in Figure 7(a). Despite the fast cooling rate (minutes to nucleate), the sizes of the particles obtained by the cooling crystallization were more than 10 times larger than the particles obtained by the freeze-drying and freeze-dissolving methods. As expected, the cooling rates in the freezing process, forming the frozen particles in the liquid nitrogen, were in a seconds time scale or even faster. The NaHCO_3_ nucleated during the same time of the freezing process or even earlier with a decrease in the solubility. There was much higher supersaturation at nucleation during the frozen process than during the cooling process. The faster nucleation rate led to much smaller particles. The confinement and limited molecules in the small droplets also limited the chance to form large crystals as in the bulk solution in cooling crystallization. In addition, there was less chance for crystal growth in the frozen particles but relatively much longer time for crystal growth in the cooling crystallization. On one hand, a large number of ice crystal molecules in the small ice area occupied the original position of sodium bicarbonate molecules, increasing the distance between the NaHCO_3_ molecules and weakening the force among the NaHCO_3_ molecules.^31,32^

Figure 7(c) shows that the NaHCO_3_ particles obtained by the freeze-drying method, the freeze-dissolving method, and cooling crystallization were the same polymorph, as the anhydrous form reported in the literature.^33^ The product obtained by cooling crystallization had a good crystalline structure with sharp peaks in its powder XRD spectrum. The powder XRD spectrum of the product obtained by the freeze-drying method revealed a semicrystalline structure, with a relatively large full width at half-maximum (fwhm). It is noted that the products obtained by the freeze-dissolving method had much smaller fwhm values, indicating a more orderly crystalline structure than the product by the freeze-drying method.

**Figure 8:**
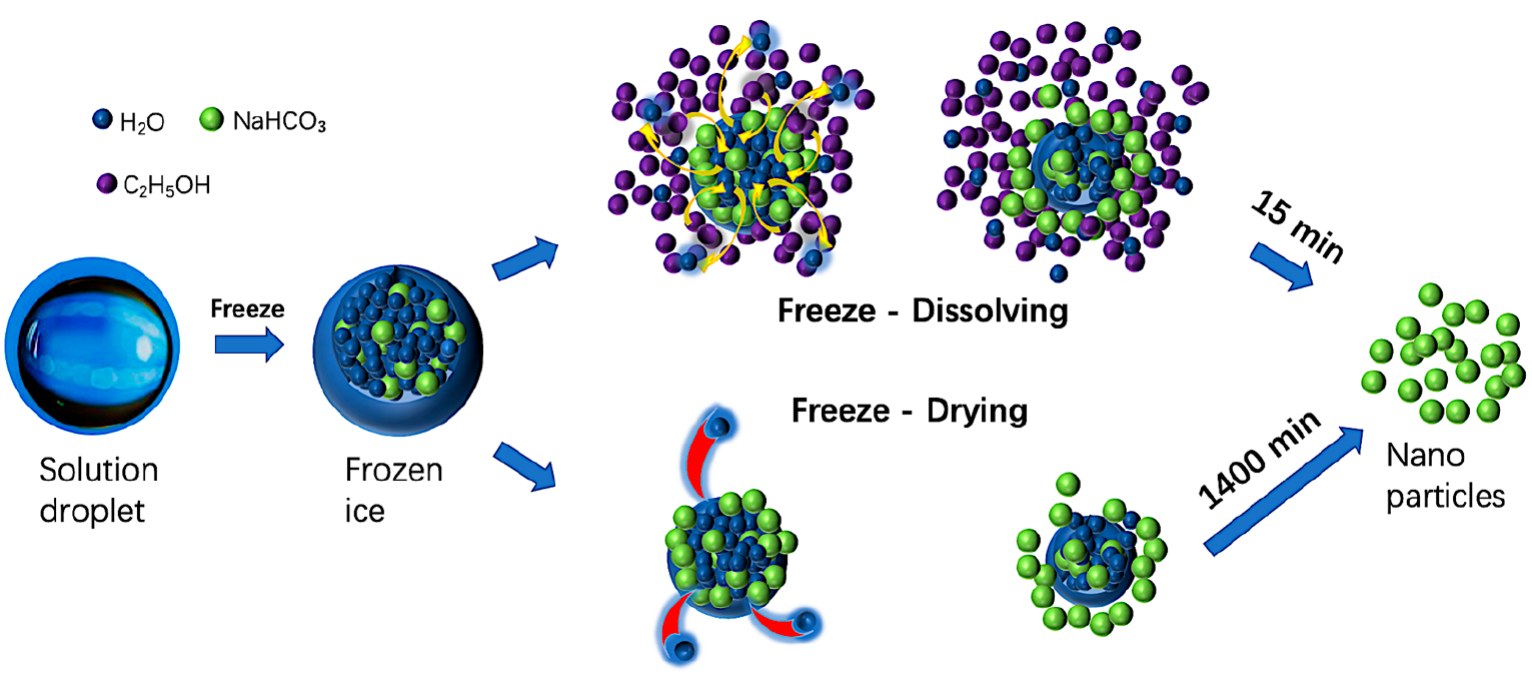
Schematic diagram of the freeze-dissolving and freeze-drying mechanisms for the formation and isolation of NaHCO_3_ nanoparticles.

Before the isolation process for both freeze-drying and freeze-dissolving methods, the poor crystalline materials formed in the freezing process were the same as shown in Figures 1 and 8. During the freeze-drying process the water molecules in the ice spherical particles sublimated to air, leaving only nanoparticles with a texture and 3D structure (some networks/fibers formed between nanoparticles) maintained. During the freeze-dissolving process, the water molecules were dissolved into the ethanol. The mixture solution of the water and ethanol had negligible solubility of NaHCO_3_, as shown in Figure 7(d). However, the very limited solubility could lead to dissolution of the amorphous or semicrystalline materials in the solution due to their thermodynamic instability and high solubility. These dissolved NaHCO_3_ molecules would recrystallize on the existing solid crystalline phase. The process could explain the reason that the particles obtained from the freeze-dissolving method had better crystalline structures. In addition, some of the 3D structure networks (or fibers) between the nanoparticles would also be dissolved based on the same principle, leading to smaller sizes of the particles obtained by the freeze-dissolving method (Figures 4 and 5).

As noted, a tiny amount of NaHCO_3_ would be dissolved during the freeze-dissolving process, and the solubility was determined to ensure sufficient materials in the frozen particles for making the nanoparticles. The solubility of sodium bicarbonate increased with the increase in temperature with same mixtures of water and ethanol, and the solubility increased with the increase the ratios of water in mixtures at the same temperature, as shown in Figure 7(d) (data in the Supporting Information). In this work, at −10 °C (263.15 K), the mass of ethanol used was seven times that of the water contained in the frozen spherical particles. The solubility of NaHCO_3_ in the mixture of water and ethanol (or with more water in the mixture) was close to 0, and therefore, the yield of nanoparticles would be very high. On the other side, a smaller amount of the ethanol could be designed to recover the particles with high yield, based on the solubility.

### Case of Ammonium Dihydrogen Phosphate

**Figure 9:**
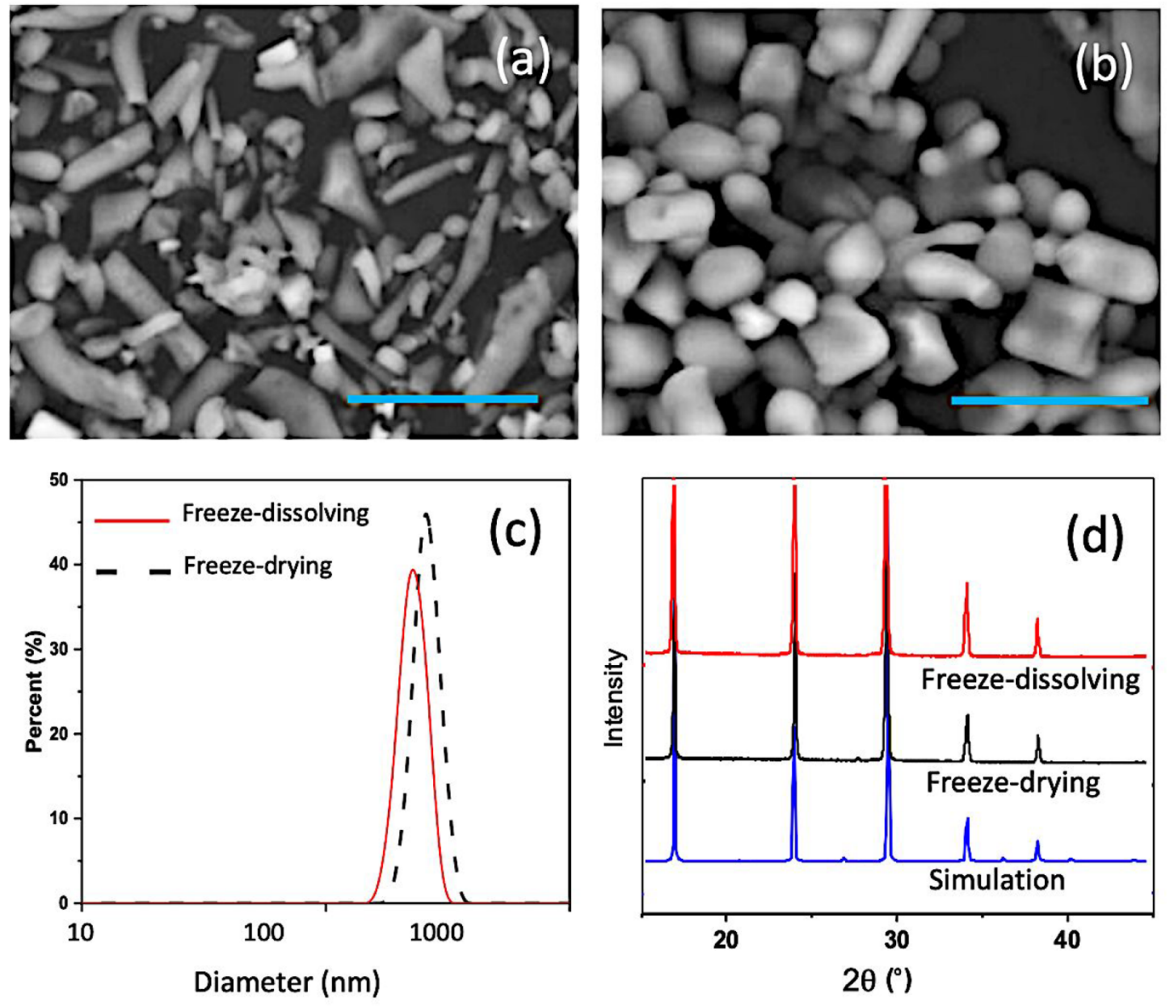
Nanoparticles of (NH_4_)(H_2_PO_4_) obtained by the freeze-drying method (a) and the freeze-dissolving method (b). Scale bar: 10 μm. Particle size distribution (c) and powder XRD spectra (d) of the particles obtained by the freeze-dissolving method (top) and the freeze-drying method (middle) and based on a single crystal structure^34^ by simulation (bottom).

The particles of (NH_4_)(H_2_PO_4_) obtained by the freeze-drying and freeze-dissolving methods were mostly below 1 μm, as shown in Figure 9(a–c). The particles obtained by the freeze-dissolving method were smaller than those obtained by the freeze-drying method, where the same frozen spherical particles were isolated by each method separately. Figure 9(a) and (b) shows that the particles of (NH_4_)(H_2_PO_4_) obtained in both methods had stick-like shapes, with some agglomerations. The powder XRD spectra of (NH_4_)(H_2_PO_4_) particles obtained by the freeze-dissolving method and the freeze-drying method proved to be the same polymorph as the anhydrate form reported.^28^ The particles obtained by the two methods had good crystalline structures, shown as sharp peaks in the powder XRD spectra. They shared the same powder XRD spectrum as that of the simulated XRD spectrum from the single crystal of the anhydrate form. The case of (NH_4_)(H_2_PO_4_) was inconsistent with the case of NaHCO_3_, showing the advantages of the freeze-dissolving method, such as smaller nanoparticles produced with lower energy consumption, a faster process, and simpler operation and facility requirements.

As the freezing process is the same for the two technologies, the efficiencies of the isolation process of the two technologies are compared. The production rate (weight/unit time) of the freeze-dissolving technology is about 100 times faster due to the fast operation time of the freeze-dissolving technology, as shown in Figures 1 and 8. The energy consumption of each operation for the two technologies was calculated based on the power, such as vacuum pump and compressor (details in Supporting Information), and the energy consumption is about 100 times less based on the g-scale products obtained in this work. We have demonstrated the products at g scale, and this method is potential to increase the batch volume for applications at much larger scale or industrial scales, and with scale-up, the time savings and energy savings of the freeze-dissolving method is expected to still be very significant.

For the freeze-drying method in similar systems as NaHCO_3_ and (NH_4_)(H_2_PO_4_), only one solvent, usually water, is used. For the freeze-dissolving method, the other solvent is usually an organic solvent, with a wide range of options, such as alcohols (ethanol used in this work), esters, and aromatic solvents. For applying this technology on producing target chemicals, compounds, or polymeric nanoparticles or ultrafine powders, two suitable solvents are required, one good solvent (high solubility for target product) with a high freezing point, *T*_GS_, and one poor solvent (low solubility) with low freezing point, *T*_PS_, in order to dissolve the frozen good solvent in the poor solvent at a dissolving temperature, *T*_d_, between the freezing points of both solvents (*T*_GS_ < *T*_d_ < *T*_GS_), allowing nanosized and microsized products to remain in the poor solvent at *T*_d_. The two cases of NaHCO_3_ and (NH_4_)(H_2_PO_4_) nanoparticles isolated by the freeze-dissolving method have demonstrated the potential wide applications of this new technology for manufacturing nanoparticles or ultrafine powder. It needs to be further investigated by optimizing the solvent compositions, solvent types, freezing process, and frozen particle sizes for producing smaller nanoparticles below 10 μm.

## Conclusions

The new freeze-dissolving method is a more efficient, more sustainable, and simpler method than the freeze-drying method to isolate nanoparticles and ultrafine powder from ice templates in frozen particles. In both methods, droplets of NaHCO_3_ and (NH_4_)(H_2_PO_4_) aqueous solutions were fast frozen to form spherical ice particles, with nanoparticles and ultrafine particles of sodium bicarbonate or ammonium dihydrogen phosphate inside the ice templates. With the freeze-dissolving method, the frozen particles were dissolved in ethanol at −10 °C to remove the ice scaffolding in 5 min. However, with the freeze-drying method, it required 1400 min to remove the ice scaffolding by sublimation. The sizes of the final products obtained by the freeze-dissolving method were smaller than those obtained by the freeze-drying method in equal experimental conditions, including the concentration of NaHCO_3_ or (NH_4_)(H_2_PO_4_) and sizes of the frozen particles. The freezing-dissolving method is about 100 times faster with about 100 times less energy consumption than the freeze-drying method as demonstrated in this work, without requirements of a vacuum and big facility. Therefore, there is potential to apply the freeze-dissolving method on a large scale with less time, less energy, and less footprint.

## Supporting Information Available

Mole fraction solubility (*x*_1_) of sodium bicarbonate in mixtures of water and ethanol from 273.15 to 313.15 K determined. Gravity method for determining the solubility. Energy consumption calculation for producing nanoparticles in this work. (PDF)
