# Supplementary material for: Freeze-Dissolving Method: A Fast Green Technology for Producing Nanoparticles and Ultrafine Powder
Source: ACS Sustain Chem Eng. 2022 Jun 9;10(24):7825–32. doi: 10.1021/acssuschemeng.2c02270 (PMC9214760; doi:10.1021/acssuschemeng.2c02270)
Supplement: Supplementary file 1 — sc2c02270_si_001.pdf [file sc2c02270_si_001.pdf]

Supporting information

Authors: Qiushuo Yu, Yingchen Wang, Jiaqi Luo, and Huaiyu Yang

Manuscript title: Freeze-Dissolving Method: A Fast Green Technology for Producing Nanoparticles and Ultrafine Powder

Number of pages: 2

Tables: 2

Table 1. Mole fraction solubility (x_1) of sodium bicarbonate in mixtures of water and ethanol from 273.15 to 313.15 K (P= 0.1 MPa)[a]

	$x_2 = 0.3237$	$x_2 = 0.2677$	$x_2 = 0.2497$	$x_2 = 0.2214$
$T(K)$	$10^4 x_1$	$10^4 x_1$	$10^4 x_1$	$10^4 x_1$
273.15	1.4731	0.9540	0.6756	0.4978
283.15	1.9698	1.1248	0.9665	0.6220
293.15	2.5305	1.6023	1.2839	1.1755
298.15	3.1486	1.8733	1.6424	1.3115
303.15	3.2697	2.0954	1.7486	1.5796
313.15	3.3874	2.4453	2.0033	1.7737

[a] x_2 is the mole fraction of water in the binary solvent mixture. Standard uncertainties (u) are u(T) = 0.05 K, u_r(P) = 0.05, $u_r(x_1)$ = 0.05, and $u_r(x_2)$ = 0.01.

Methodology for Solubility determination

Excess sodium bicarbonate powder was added to a glass vial containing solvent, and was stirred with a magnetic stirrer bar for 5 h to achieve solid-liquid phase equilibrium in a constant temperature. A 10 ml of supernatant was filtered through 0.22 μ filter into a pre-weighed vial to obtain saturated solution. The total weight of vial was measured and then the vial was dried completely at 50 ℃. The mole fraction solubility (x_1) of sodium bicarbonate in the mixed solvent was calculated by Eqn (1), and the mole fraction of water (x_2) in the mixed solvent was calculated by Eqn (2):

$$x_1 = \frac{m_1/M_1}{m_1/M_1 + m_2/M_2 + m_3/M_3} \quad (1)$$

$$x_2 = \frac{m_2/M_2}{m_2/M_2 + m_3/M_3} \quad (2)$$

where m_1, m_2, and m_3 represent the mass of the solute, water, and ethanol, respectively. M_1, M_2, and M_3 are their corresponding molecular weights.

Table 2. Estimation of the energy consumption for freeze-dissolving and freeze-drying technologies with products in order of g scale

Freeze - drying			Freeze - dissolving		
Operation	Power	Time	Operation	Power	Time
Vacuum pump	400 w	1400 m	Vacuum pump for filtration	400 w	5 m
Compressor	100 w	1400 m	Water bath compressor	200 w	5m
			Water bath pump	200 w	5 m
			Drying oven	500 w	5 m
Total energy consumption：	42×10^6 J			390×10^3 J	
